# The Late Sir Richard Quain

**Published:** 1898-03-19

**Authors:** 


### The Late Sir Richard Quain.

The death of Sir Richard Quain removes a very conspicuous personality from the world of medicine. Born in 1816, he entered the profession in or about 1840, and, having been for five years house surgeon or house physician to University College Hospital, he engaged in private practice as soon as he relinquished the last-mentioned appointment. He was very speedily successful, and thus, for more than fifty years, has been holding a high place, and for the last twenty of them a place not far removed from the highest, among the physicians of the metropolis. His work as a practitioner or consultant, arduous and extensive although it was, displayed only one aspect of his many-sided character, and he was as familiarly known to the leaders of pleasure-loving London as he was to his patients, or to his colleagues at the Brompton Hospital. Few men have possessed in a greater degree the art of making friends; none have ever been more loyal to them when they were made. "An Irishman of the best type," as the *Times* happily described him, he combined the hearty joviality of his nation with a steadiness of application which has seldom been surpassed by the most prosaic or the most ponderous philosopher, an application which enabled him to be mainly instrumental in convincing England of the necessity of "stamping out" the cattle plague, and which, at a more recent period, was rendered evident by the production of his "Dictionary of Medicine." His qualities as a man of business were conspicuous in the Medical Council, to which he was appointed as a Crown representative in 1863. In the following year he became Treasurer, and held that office until his election as President in 1891. He was always an active member, and for many years Chairman, of the Pharmacopœia Committee, and, at the unanimous request of his colleagues, consented to retain his Chairmanship after he became President. The new Pharmacopœia recently sanctioned by the Council, and soon about to be issued, will be largely indebted for its merits to his sagacity and experience; and it is an open secret that he would have resigned his seat on the Council some months ago, under the pressure of the malady which has at last proved fatal, had it not been for his desire to see the completion of a work which he had done so much to promote. When he was unable to leave his house, some of the meetings of the Committee were held there; and he had at last the satisfaction of being able, as Chairman of the Committee, to hand over the volume in a completed state to the Council, and, as President of the Council, to sign the official report to the Treasury upon the subject, leaving it to them, as the Act requires, to fix the selling price of the book. His urbanity as President was the most effectual possible solvent of the difficulties sometimes incidental to discussions among men warmly attached to different opinions; and his memory of past occurrences and past debates was at once accurate and unfailing. During his Treasurership, he would often arrest the eloquence of a lengthy speaker by a playful reminder of the expense of the sitting, and of the amount of money which his words were costing per minute; and this would be done with a perfection of good temper which rendered it impossible to be offended with him.

Soon after the meeting of the Council in May, 1897, Sir Richard was found to be the subject of calculus, and he submitted twice, if not three times, to the operation of lithotrity. For a while all seemed to be well—he saw his patients, dined with his friends, talked cheerily about his condition. But, long before October, a melancholy change became apparent in his condition. He began to suffer from intestinal obstruction, only too clearly due to the presence of a malignant growth, and colotomy was performed in the left iliac region. From this time he scarcely ever left his bed, except to go to and to return from a house which he rented at Putney during the latter part of the summer. He was a great lover of dogs, and had a favourite little terrier, which was his constant companion in his carriage. She died at Putney; and, before returning home, Sir Richard had himself carried down to see the place in the grounds where she was buried. When he reached Harley Street, most of those who were admitted to him expected an earlier termination of his illness; but his wonderful vitality struggled against pain and weakness in an extraordinary manner, and his intelligence, and his interest in affairs, remained unabated almost to the end. It was not until last week that he became unable to receive short visits from his most intimate friends, or that he ceased to inquire about medical questions and the business of the Council. At last, on the morning of Sunday, the 13th instant, he passed peacefully away, and he was buried at Hampstead on Wednesday in the grave to which Lady Quain had preceded him.

